# Developing Nursing Standard Guidelines for Nurses in a Neonatal Intensive Care Unit: A Delphi Study

**DOI:** 10.3390/healthcare8030320

**Published:** 2020-09-04

**Authors:** Hanna Lee, Da-Jung Kim, Jeong-Won Han

**Affiliations:** 1 Department of Nursing, Gangneung-Wonju National University, Gangwon-do 26450, Korea; hannalee@gwnu.ac.kr; 2 College of Nursing Science, Kyung Hee University, Seoul 02447, Korea; jung-321@hanmail.net

**Keywords:** education, Delphi technique, infant, nursing

## Abstract

The purpose of this study is to develop nursing standard guidelines for nurses in a neonatal intensive care unit. The Delphi method was used in this study to elicit expert consensus. Thirteen experts who were nurses and pediatric adolescent specialists working in the neonatal intensive care unit participated in the study. In this study, 178 items were developed based on 5 nursing practice standards and 7 standards of professional practice. An additional 10 items were included based on observation in the neonatal intensive care unit. After expert validation, a final total of 184 items was developed. The standard guidelines for high-risk neonatal care developed in this study for practical clinical education in nursing are significant because they reflect the nursing practice standards in Korea and characteristics of nursing practice in the neonatal intensive unit.

## 1. Introduction

Protecting human health is a goal for all healthcare practitioners, and high-risk infants especially require greater care than other populations. This group is broadly defined as including: (1) the preterm infant; (2) the infant with special healthcare needs or dependence on technology; (3) the infant at risk because of family issues; and (4) the infant with anticipated early death [1]. The proportion of preterm infants who also have a low birth weight, defined as less than 2.5 kg, has increased steadily over time, rising from 3.3% in 1997 to 4.7% in 2007 and reaching 6.2% in 2018, with concomitant increases in high-risk infants—those categorized as in need of the neonatal intensive care unit (NICU) in South Korea (hereafter Korea) [2]. Immediately after birth, high-risk infants are admitted to the NICU, where they spend at least 24 h in the care of NICU staff, who play a critical role in their survival and eventual thriving [3]. NICU nurses provide various treatments and nursing care to newborns, including oxygen therapy, mechanical ventilation, maintenance of vital signs, nutritional supplementation, and infection prevention [4]. Notably, after birth, most newborns in the NICU experience various respiratory issues while transitioning to voluntary breathing, which may lead to emergency situations. In these situations, emergency nursing care from experienced nurses is also vital [5]. Moreover, NICU nurses also facilitate the interaction between the newborns and their parents and help the parents to learn their new parental roles [4]. Clearly, NICU nurses face the burden of achieving expertise in a range of complicated tasks spanning several domains in order to respond to the rapidly changing health conditions of newborns, and they are required to have excellent clinical decision-making skills to provide timely, accurate nursing care depending on the newborns’ conditions [6]. Clinical decision-making skills in NICU nurses are one of those nursing competencies that increases the quality of nursing and exerts a positive influence on the treatment outcome in newborns [7].

It is also vital for the nurses to have standard guidelines for practice based on these assessments [8]. In clinical situations, standard guidelines help to improve nurses’ competency. Moreover, in light of most Korean nursing students’ limited clinical experiences with high-risk infants, such guidelines can be used as educational materials, providing systematic knowledge of this highly specific area of nursing [9].

At present, little work has been done on this issue in the Korean context. Jun (2000) [10] analyzed the workload of NICU nurses and divided their tasks into 16 direct nursing activities and 7 indirect activities. These activities, however, were categorized based on nursing tasks performed in specialized units other than NICU, as well as in more general units. Park (2007) [11] categorized neonatal nurse practitioners’ tasks into 6 roles, 12 duties, and 46 tasks comprising 315 activities. However, with the exception of the management of high-risk infants in delivery rooms, the duties investigated by Park (2007) [11] involve care for healthy newborns. Moon (2006) [12] classified NICU nurses’ tasks into 42 nursing behaviors for billing purposes, but the scope of tasks of NICU nurses was underestimated in this study. In terms of previous studies conducted in other countries, a study that developed neonatal nursing standards for Cambodia included five stages of nursing, health education, health promotion, continued education, and communication, but these are more limited compared to the range of tasks performed by Korean NICU nurses [8].

Because some Korean hospitals offer only limited pediatric clinical placements for nursing students so as to prevent infections in newborns, some students graduate without any clinical exposure to the NICU [13]; this makes it difficult for students to cultivate professional competencies through hands-on practical experience. When nursing students graduate to become new nurses without sufficient clinical exposure to the range of unique situations which occur in the NICU, they cannot respond to emergency clinical situations as well as if they had practical experience, and this is directly related to patient safety. In particular, nurses who lack experience detecting and quickly responding to emergency situations have been reported to delay emergency responses with high-risk infants [14]. Moreover, these nurses have been found to have a higher desire to change jobs due to decreased self-efficacy at work [15]. Therefore, in order to train competent nurses in an environment where nursing education for high-risk infants is limited, and to retain these nurses and facilitate their professional growth, it is necessary to develop standard guidelines on the tasks of NICU nurses and to use these guidelines in nursing education to form a foundation for practice.

To this end, the present study aimed to develop standard guidelines for NICU nurses and to obtain expert opinion on these guidelines in an attempt to provide basic data in clinical practice and to create evidence-based, targeted, relevant educational materials for nursing students to improve both their practice and their experience.

## 2. Materials and Methods

### 2.1. Study Design

The present study is a methodological study which aims to understand the tasks of NICU nurses and to develop standard guidelines for their practice and provision.

### 2.2. Study Participants

The main criterion for the selection of experts on the 13-member panel was to include people who had been recognized for their professional knowledge and experience. The criterion was operationalized as follows: (1) Those who had worked in the neonatal intensive care unit for more than 5 years, and had been active in medical institutions to date (2) Those who had undergone a neonatal intensive care course and had experience in developing and publishing books or papers related to neonatal intensive care (3) Persons with experience in developing related policies, educational programs, and conducting research as executives of neonatal intensive care-related organizations (4) neonatal intensive care workers of government or private organizations who had experience in developing and participating in related projects.

### 2.3. Study Stages

**Figure 1:**
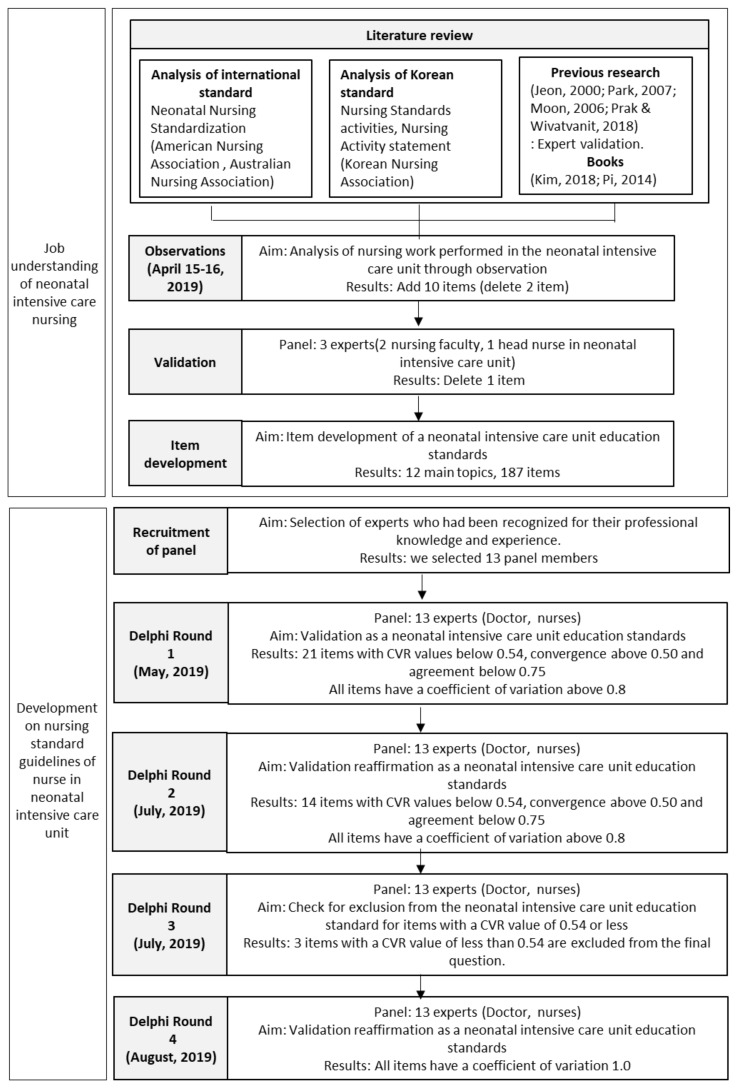
Process of the study.

The process of this study is shown in detail in Figure 1.

#### 2.3.1. Understanding the Job of Neonatal Intensive Care Nursing

##### Literature Review

The researchers reviewed the literature on standard guidelines for NICU nurses between 1 March and 15 April 2019. This literature review comprised a review of neonatal nursing standards suggested by the American Nurses Association (2004) [16] and the Australia College of Nursing (2019) [17], nursing standards from the Korean Nurses Association (2003) [18], a pediatric nursing textbook [19], a high-risk infant nursing textbook [20], previous studies on neonatal nursing [10,11,12], and job descriptions from hospitals.

##### Observations

At this stage, the researchers found it important to conduct a clinical field observation and a perusal of nursing records to see if any relevant tasks were overlooked during the literature review and job description. The researchers also investigated nursing work performed by NICU nurses through observation. For clinical observation, the researchers visited the director of nursing service at a general hospital located in G Province, explained the purpose of the study, and obtained approval for observation; the clinical observation was conducted on 15 and 16 April 2019. The observer was one trained individual who has a high understanding of the clinical practice being observed and nursing education, has completed a PhD in nursing, provided clinical lectures and training for pediatric nursing, and has nine years of clinical experience including experiences in NICU and pediatric settings. The observees were nurses who had at least 10 years of clinical experience with at least five years of experience in the NICU; observees were selected through consultation with the head nurse. Consent was obtained from all observees. Two nurses working in day and afternoon shifts on April 15 were observed, and one nurse working in the night shift on April 16 was observed. Moreover, in order to confirm whether any relevant items were omitted during observation, nursing records through the electronic medical system in the hospital were reviewed at the end of the observation periods.

##### Validation

The content validity of the items identified through clinical observation and literature review was confirmed by two nursing professors and one NICU head nurse working at a tertiary hospital in S City. The content validity in the expert panel was analyzed based on the content validity ratio (CVR) suggested by Lawshe (1975) [21].

#### 2.3.2. Development of Nursing Standard Guidelines in the Neonatal Intensive Care Unit

##### Recruitment of the Panel of Experts

No clear rule exists for the number of panel experts and selection criteria in a Delphi study. However, because a Delphi study depends on expert opinions rather than direct data, the composition of the expert panel is crucial for its successful application. The previous studies suggested that at least 10 experts are needed and that small groups of experts (10 to 15) may yield useful findings [22,23]. The expert panel candidates in this study were NICU nurses working at three tertiary hospitals in S City and G Province in Korea, each of whom has more than five years of experience in the field, as suggested by Benner (1984) [24]. The following criteria were applied to select the panel nurses: those who are members of the Korean Association of Neonatal Nurses, have experience in conducting research on neonatal intensive care, and have completed an NICU nursing program; those with master’s or PhD degrees in neonatal intensive care; those who are working as lecturers on neonatal intensive care in nursing programs at universities; those who are licensed to provide specialized care for pediatric and neonatal populations; and those who have experience drafting job descriptions at hospitals. Moreover, we also selected specialists responsible for neonatal intensive care at tertiary hospitals who have more than four years of experience in addition to research experience on neonatal intensive care and pediatrics. In total, 14 candidates were identified for possible inclusion in the study panel. The researchers reviewed the candidates’ clinical experience in NICU and activity in expert groups and evaluated their independence, adequacy, quality, and responsibility. Of these 14, one nurse who had more than five years of experience in NICUs was excluded, as this person had moved to an adult unit more than two years ago. Finally, we selected 13 panel members.

##### Delphi Round 1

This study’s Delphi survey was conducted between 30 May and 31 August 2019. In the first Delphi round, preliminary items for the standard guideline for NICU nurses identified through clinical observation and literature were presented, and the experts were asked to rate each item on a 5-point Likert scale. In the first Delphi round, in order to account for the experts’ various opinions, the survey questions asked the experts to describe the work of NICU nurses; based on this information, items could be revised, deleted, or added.

##### Delphi Round 2

The second Delphi round was designed based on the results of the first Delphi round. The researchers analyzed the results of the first Delphi round and provided the mean, standard deviation, and inter-quartile range for each item in the second-round questionnaire, along with the responses of each expert in the first Delphi round. Through this process, the experts were able to compare their own responses to those of the other participants. This second Delphi round provided opportunities for the experts to reflect, revisit, and revise their opinions by comparing their own responses to the mean of all experts. If the experts’ opinions deviated outside the inter-quartile range of the mean response of all experts, the experts were asked to describe why they evaluated the items as such. CVR, convergence, agreement, and coefficient of variation were calculated in the second Delphi round, as in the first Delphi round.

##### Delphi Round 3

The third Delphi round aimed to reach a consensus on nursing work standards based on the results of the first and second Delphi rounds. The results of the first and second rounds were analyzed, and the experts were asked whether the items with a CVR of and below 0.54 should be excluded or retained in the NICU nursing work standards, along with their reasoning for this response.

##### Delphi Round 4

In the fourth Delphi round, the NICU standard guideline developed through the third Delphi round was confirmed for content validity. Moreover, when clinical experts had opinions on the NICU nursing work standards, they were asked to provide these opinions.

### 2.4. Ethical Considerations

The present study was reviewed by the institutional review board of C University in Korea (1040271-201902-**-037), and the purpose of the study was explained to the participants prior to obtaining their written consent. The written consent outlined voluntary participation, anonymity, and scope of use of the results. The participants were reminded that they may withdraw their participation whenever they wished and that the study results will be used solely for research purposes. Consultation fees were provided to the expert panel after each round.

### 2.5. Data Analysis

#### 2.5.1. The Positive Coefficient of Experts

The positive coefficient of experts is the effective recovery rate of the expert consultation questionnaire and can reflect positive input from the experts [25].

#### 2.5.2. The Degree of Expert Authority

The degree of expert authority was determined by two factors.

First, that there is the expertise to make a judgment on the consultation, this represents the ability of the expert to judge the content of the coefficient (CA). A CA sum equal to 0, suggests that the impact of expert judgment is small, A CA sum equaling 0.5, means the influence degree of expert judgment is medium, and finally, a CA sum equaling 1.0, indicates that the influence degree of expert judgment is great. In this study, the experts used the terms “working experience,” “theoretical analysis,” “literature,” and “intuitive sense” as the basis for judgments.

Second, the degree of familiarity of the experts on the inquiry content. (CS) [26] A Likert scale was used; very familiar (1 point), largely familiar (0.75 point), generally familiar (0.5 point), less familiar (0.25 point), and unfamiliar (0 point). The degree of familiarity of each expert was calculated statistically.

The degree of expert authority (CR) the coefficient (CA) and the degree of familiarity of the inquiry content (CS), were calculated thus: CR = (CA + CS)/2. Values greater than 0.7 were considered to be acceptable [27].

#### 2.5.3. Degree of Expert Coordination

Based on this, the criteria for content validity was set as a CVR of 0.54, and items with a CVR at, or above 0.54 were considered to have adequate content validity. Moreover, the criterion for convergence was set below 0.50, and for agreement at 0.75 and above [28]. To achieve stability of items through expert agreement, coefficients of variation were calculated, and those with a coefficient of variation exceeding 0.8 indicated a need for re-evaluation [29].

## 3. Results

### 3.1. General Characteristics of the Delphi Survey Expert Panel

The panel comprised 1 male (7.7%) and 12 female (92.3%) experts; participants included 1 physician (7.7%) and 12 nurses (92.3%). The mean age of the experts was 33.3 years, and the experts had an average 10.8 years of experience. Moreover, the experts had an average of 9.7 years of experience in the NICU. In terms of special licenses, three experts were International Board-Certified Lactation Consultants. The 13 experts thought that 5.4 years of clinical experience on average were necessary to achieve expertise as an NICU nurse (Table 1).

### 3.2. Positive Coefficient of Experts

In the first, second, third and fourth round of Delphi expert consultation, the recovery rate of the four rounds of expert inquiry questionnaires was 100%, and the effective rates were 100%.

### 3.3. The Degree of Expert Authority

The average CA = 0.78 and CS = 0.94 for the research experts. The average was CR = 0.86 for all the experts in this study.

### 3.4. Development of the Standard Guideline for NICU Nurses

In order to select items for the standard guideline for NICU nurses, this study first selected 178 preliminary items based on previous studies conducted on nursing standards in the NICU [10,11,12,18,19,20]. The selected items were classified based on the Korean Nurses Association after consideration of nursing education in Korea; nursing work standards comprised five domains, and professional performance standards comprised seven domains (Table 2). Of the items observed in clinical practice, 12 items did not overlap with those found through the literature review and were thus included in the 190 preliminary items for the standard guideline. The content validity of the selected items was confirmed by two nursing professors and one head nurse working at the NICU of a tertiary hospital in S City. Blood sampling and foot stamping, which had content validity below 0.5, were excluded from the 12 items found through clinical observation; the other 10 items, including foley catheter insertion, enema, phototherapy, surgical patient care, umbilical cord care, and G-tube feeding, had CVRs of 1.0 and were retained. Moreover, shun syndrome, which was identified through the literature review, had a convergence below 0.5, suggesting that it does not reflect the Korean nursing environment. It was thus excluded. As a result, a total of 3 items were excluded from the initial 190 items; the remaining 187 items were selected for potential inclusion in the NICU standard guideline. In the NICU nursing standard guideline, the nursing work standard domain comprised data collection, diagnosis, planning, performance, and evaluation. This was based on the standard nursing activities outlined by the Korean Nurses Association.

### 3.5. Delphi Survey for the NICU Nursing Standard Guideline

The results of the first and second Delphi rounds are shown in Appendix A. In the first Delphi round, 21 items had CVRs below 0.54, a convergence of 0.50 or above, and agreement below 0.75, but the items had coefficients of variation above 0.8. In the second Delphi round, 14 items had CVRs below 0.54. The third Delphi round investigated whether it would be valid to exclude those 14 items; ultimately, 3 items with CVRs below 0.54 were excluded from the final guideline. The excluded items were: observe facial expression of mother/caregiver to identify feeling; demonstrate the use of manual breast pump; body weight and condition of the mother before and after delivery. A total of 184 were identified through the third Delphi round.

In the fourth Delphi round, all items had CVRs of 1.0. When asked for input on the standard guideline for NICU nurses developed in this study, the experts emphasized that systematic education should be provided in schools and in clinical practice based on the standard guideline. In particular, the experts made the following statements:

“In real life, students can’t touch the babies during their clinical placement. The caregivers react sensitively to students touching the babies… so students can’t really do anything. When we accept new nurses, we expect them to know the basics, such as blood pressure, body temperature, and weight measurement…”

(Expert 1)

“I hope the new nurses will have sufficient knowledge of normal and abnormal from their training in school. We can’t provide theoretical training in clinic due to a lack of time. In clinic, we have to provide care based on what we learned in school, so we should be well aware of the details.”

(Expert 2)

## 4. Discussion

The present study was intended to develop a standard guideline for NICU nursing through the Delphi method and to discuss future directions which may allow nursing students to obtain expert knowledge and skills in neonatal intensive care despite the limited clinical placements available to trainee nurses.

First, when experts on the panel offered their opinions on the proposed NICU standard guideline, they noted that they expected student nurses to be educated on basic nursing tasks, such as assessment, nursing diagnosis, planning, and performance, rather than professional tasks, such as evaluation, education, evidence-based nursing, cooperation, use of resources, and quality management. Recently, clinical experiences in nursing care for high-risk infants have been limited due to details of the clinical situations in Korea and the need to limit infection potential among high-risk newborns; therefore, nursing students have had little clinical exposure in the NICU [13]. To address this issue, it is crucially important for nursing educators to have access to basic data to prepare educational materials. In particular, clinical experts on the research panel emphasized that students should gain sufficient knowledge of nursing for high-risk infants at school. At a minimum, the experts agreed that student nurses should be able to identify normal and abnormal conditions in high-risk infants during any clinical placement, as it is often difficult to provide theoretical education in clinical situations. The main opinion of clinical experts was that there were no practical tasks that students could do in the current neonatal intensive care unit practice environment, so they wanted to sufficiently practice physical assessment and some technical skills in school. In particular, there is a lack of time to impart theoretical knowledge during clinical postings, which is why it is necessary to sufficiently learn theory in school and, based on that knowledge, observe clinical cases. The panel of experts also emphasized the importance of infection control and expected NICU nurses to learn about it thoroughly. On the other hand, aspects of NICU nursing that involve different systems and are hospital-dependent such as respiratory equipment, monitor operation, and hospital discharge, are expressed as areas that need to be strengthened in clinical education in the future. Further, the experts also suggested that schools should adopt other learning strategies to support students’ skill development. For instance, although physical assessment is a basic task that is important for all nurses, students cannot directly touch newborns in clinical settings. Therefore, schools should strengthen objective structured clinical examination (OSCE), clinical performance/practice examination (CPX), standard patient (SP), and simulation education [30]. In addition, it can be seen that it is necessary to develop education programs including various scenarios, and to evaluate each one. This finding coincides with a previous study that applied virtual reality and blended simulation of asthmatic children [31] and found that various simulation systems, including high fidelity simulation, can improve nursing students’ critical thinking, problem-solving, and clinical performance. On this basis, nursing educators should design clinical education programs which employ various evidence-based learning strategies when developing material on nursing for high-risk infants. Panel experts also pointed out the need for nursing education on the risks of infection, hypothermia, impaired gas exchange, and neonatal asphyxia, which are being emphasized in clinics, in addition to physical assessment. This aligns with previous studies emphasizing the importance of developing targeted scenarios and educational programs, such as neonatal CPR emergency nursing programs [32], simulation scenarios for apnea emergency management [33], and emergency airway management programs for NICU nurses [34] in order to improve the clinical competency of students. As most Korean studies have focused on developing scenarios for respiratory conditions, such as dyspnea and CPR, future studies should develop various cases and scenarios targeting areas of particular concern to the expert panel, such as hypothermia, impaired gas exchange, neonatal asphyxia, and risks of infection. These studies should provide education on using these materials and evaluate their effects on students’ competencies. In addition, in order to meet student nurses’ learning goals, future studies must consider the appropriate means of delivery for such scenarios.

Second, as a result of this study, the nursing standard guidelines for nurses in a neonatal intensive care unit consisted of nursing practice standards and professional performance standards. The professional performance standards comprise ethics, performance evaluation, education, evidence-based practice and research, consultation, and operation, proper use of resources, and quality management. In contrast to a previous study conducted outside of Korea on nurses providing care for newborns [8]—which divided neonatal nurses’ work standards into simple nursing stages and overlooked nursing practice domains—the present study has developed the proposed NICU standard guideline based on the nursing practice standard developed by the Korean Nurses Association, by applying professional performance standards particularly suitable for Korea. Another previous study conducted in Korea divided NICU nurses’ work into direct and indirect nursing activities while focusing only on nursing work standards [10]. The present study also overcomes this limitation, adding items identified through clinical observation to reflect recent changes in nurses’ work. The present study once again confirms that Korean nurses providing neonatal intensive care are required to perform a range of tasks from simple to highly complex, in addition to a range of emergency, professional, and educational activities and roles. As these competencies are not developed in a short period, it can be seen that a systematic step-by-step, education program is needed from the undergraduate level up to higher levels. According to a recent study on the ethical environment of neonatal intensive care unit nurses which targeted Korean nurses, neonatal intensive care unit nurses have higher levels of moral anguish than other departments, leading to higher work stress and turnover rates. The study suggests that education is necessary to overcome this problem [35]. Therefore, in the neonatal intensive care training course, in addition to theoretical education, case-based learning related to professional performance standards is required.

Third, the items excluded from the present study mostly concern breastfeeding. The results suggest that limited breastfeeding and tube feeding are more common in Korea, in contrast to other countries where breastfeeding is often provisioned in the NICU. In Korea, the mothers are trained on breastfeeding in postpartum care facilities and obstetrics clinics; it is, therefore, appropriate to exclude these tasks from an accounting of NICU nurses’ main activities. Other tasks related to public and community engagement, such as “assess whether the family is able to take care of financial issues,” had low CVRs in the first and second Delphi rounds. This may be due to the study context, as financial support and community engagement for high-risk infants are provided through administrative and clinical cooperation departments in Korea. However, the third Delphi round revealed that experts considered financial support assessment for high-risk infants and their families a part of NICU nursing professionals’ repertoire. Bearing this in mind, future studies should investigate the roles of Korean NICU nurses in relation to specific professional performance domain items.

## 5. Conclusions

The present study used the Delphi method to develop a NICU nursing standard guideline for the Korean context and to confirm aspects of practice and professional skills that should be emphasized in clinical education for nursing students. This study is significant in that it developed a NICU nursing standard guideline upon consideration of the Korean clinical environment through clinical observation and literature review. Moreover, the proposed standard guideline can also be used as basic data when developing clinical guidelines for nurses in practice. Future studies should select specific domains and skills from the NICU nursing standard guideline that are expected to be demonstrated in practice by clinical experts, developing educational standard guidelines and materials based on these target areas.

## 6. Limitations of the Study

The present study is limited in that only clinical experts’ opinions were considered in selecting the items assessed and ultimately included or excluded from the guidelines, determining which aspects were important to clinical placement and education on nursing for high-risk infants. To expand the relevance and applicability of this work, future studies should consult students who have had clinical placements in order to identify knowledge and techniques contained within the NICU nursing standard guideline that are difficult to obtain in clinical settings. This information can be used to develop and apply targeted educational programs to improve both nursing students’ experience and professional standards of care in practice.

## Figures and Tables

**Table 1:** Participant information.

Variables	Category	*n*	%
Sex	male	1	7.7
	female	12	92.3
Occupation	physician	1	7.7
	nurses	12	92.3
Age (yrs.)	<30	3	23.1
	30~35	8	61.5
	36~40	1	7.7
	<40	1	7.7
Total clinical experience (yrs.)	5~10	6	46.1
	11~15	5	38.5
	<15	2	15.4
Experience in the NICU (neonatal intensive care unit) (yrs.)	5~10	7	53.8
	11~15	5	38.5
	<15	1	7.7
International Board-Certified Lactation Consultants	Yes	3	23.1
	No	10	76.9
Clinical experience to gain expertise as a NICU nurse, considered by experts(yrs.)	<5	2	15.4
	5~9	10	76.9
	≦10	1	7.7

**Table 2:** Korean Nursing Association’s nursing standards.

Domain	Category	Explanation	Example
**1. Nursing practice standard**	**Data collection**	Collect data related to the subject’s health	Collect data according to the patient’s health status or the urgency of their needs Revise and supplement the data collected through continuous interaction with individuals, families, communities and related health care professionals.
	**Diagnosis**	Analyze the collected data and make a nursing diagnosis	Analyze the collected data Make nursing diagnosis based on data analysis
	**Plan**	Establish a nursing plan necessary to achieve the target’s nursing goals	Set nursing goals based on nursing diagnosis Establish a nursing plan with continuity of nursing
	**Performance**	Carry out nursing intervention according to the nursing plan	Nursing arbitration based on nursing plan Carry out nursing intervention, including health promotion and maintenance, disease prevention
	**Evaluation**	Assess nursing intervention	Continuously evaluate nursing Involve caregivers, families, and health teams in the evaluation process
**2. Professional performance standards**	**Ethics**	Conduct all nursing activities and decision-making ethically from the subject’s point of view	To protect the right of subject’s autonomy and dignity Maintain a trust relationship with the subject
	**Performance evaluation**	Conduct all nursing activities and decision-making ethically from the subject’s point of view	Participate in peer review activities The results of work performance evaluation and peer evaluation are reflected in job improvement
	**Education**	Acquire and maintain the latest knowledge and skills	Continuous participation in educational activities to acquire the latest nursing knowledge and skills Accumulate work experience to maintain the latest practical skills and abilities
	**Evidence-based practice and research**	Conduct research and use the results in practice	Conduct research to develop nursing practice Conduct joint research with other academics
	**Consultation** **and cooperation**	Support colleagues and cooperation with health teams	Communicate with colleagues and health teams Create a supportive work environment
	**Use of resources**	Utilize resources necessary for nursing work	Identify human and material resources Evaluate and utilize the stability and efficiency of resources
	**Quality management** **in nursing**	Participate in quality management activities according to the working environment	Identify indicators to be used for monitoring the quality and effectiveness of nursing Participate in a multidisciplinary quality assessment team

## Appendix A

**Table A1:** Nursing Standard Guidelines of Nurse in Neonatal Intensive Care Unit.

Items		1st Median	1st CVR	1st Coefficient of Variation	2nd Median	2nd CVR	2nd Coefficient of Variation
**A. Assessment**							
**1. Physical assessment**							
Chief complaints: most serious symptoms/signs of illness-causing neonate to hospital		5.00	1.00	0.06	5.00	0.00	0.00
Check temperature, airway, breathing, and circulation (TABC)		5.00	1.00	0.06	5.00	1.00	0.00
*Assess growth status*							
* Weight in gram		5.00	1.00	0.11	5.00	1.00	0.10
* Length		5.00	0.69	0.17	5.00	0.85	0.15
* Head circumference		5.00	1.00	0.11	5.00	1.00	0.09
*Evaluate general appearance*							
* Level of consciousness: state of alertness		4.00	0.69	0.17	4.00	0.85	0.15
* Skin color: integrity and perfusion		5.00	1.00	0.09	5.00	1.00	0.09
* Activity: range of spontaneous movement		5.00	1.00	0.11	5.00	1.00	0.11
* Postures: muscle tone		5.00	1.00	0.10	5.00	1.00	0.10
*Obtains maternal history*							
* Apgar score		5.00	1.00	0.10	5.00	1.00	0.09
Gestational age		5.00	0.85	0.13	5.00	1.00	0.08
Mode of delivery		5.00	0.69	0.17	5.00	1.00	0.09
Medications used and feeding provided		5.00	1.00	0.10	5.00	1.00	0.09
*Assess skin integrity, muscle, and skeleton*							
Skin color		5.00	0.85	0.13	5.00	1.00	0.09
Skin condition: rashes, pustule, peeling, plethora, dry, erythema, infected, edema, and injury		5.00	1.00	0.06	5.00	1.00	0.00
Muscle tone: spontaneous movement		5.00	1.00	0.09	5.00	1.00	0.09
Jaundice		5.00	1.00	0.06	5.00	1.00	0.06
*Check head, face, and neck*							
* Head: shape, size, scalp		5.00	1.00	0.11	5.00	1.00	0.06
* Fontanelles: sutures		5.00	1.00	0.11	5.00	1.00	0.08
* Eyes: size, position structure		4.00	0.69	0.25	4.00	0.85	0.15
* Nose: position structure		4.00	0.54	0.26	4.00	0.69	0.17
* Mouth: palate, teeth, gums, tongue, frenulum, jaw size		5.00	1.00	0.11	5.00	1.00	0.09
*Assess chest and respiratory system*							
Chest: size, shape, symmetry, movement, breast tissue, and nipples		5.00	0.85	0.13	5.00	1.00	0.06
* Respiratory system: lung sounds, signs of respiratory distress, breathing pattern, oxygen, needs, level of FiO2, and SpO2 and chest retraction		5.00	1.00	0.06	5.00	1.00	0.00
*Assess cardiovascular system*							
Heart rate/sounds		5.00	1.00	0.10	5.00	1.00	0.06
Pulse/femoral pulse and rhythm		4.00	0.85	0.15	4.00	0.85	0.15
Blood vessels		4.00	0.69	0.17	4.00	1.00	0.10
* Blood pressure		5.00	0.85	0.11	5.00	1.00	0.08
*Assess abdomen and gastrointestinal system*							
* Abdomen: size, shape, symmetry, palpate live, spleen, and kidneys		4.00	0.69	0.17	4.00	0.86	0.15
* Abdominal condition: soft, firm, redness, mass, and lobe visible		5.00	1.00	0.08	5.00	1.00	0.06
* Umbilicus: bleeding, discharge, detached, and smell		5.00	1.00	0.11	5.00	1.00	0.06
Breast feeding/feeding frequency: sucking		5.00	1.00	0.09	5.00	1.00	0.06
* G-tube feeding, Artificial feeding		5.00	1.00	0.08	5.00	1.00	0.06
Bowel movement: meconium or stool condition/color, vomiting, nausea		5.00	1.00	0.06	5.00	1.00	0.00
*Assess genitourinary*							
* Abnormality: open passage for urine and stool, any discharge		5.00	0.85	0.13	5.00	1.00	0.08
* Anal position/imperforate		5.00	1.00	0.10	5.00	1.00	0.06
*Assess neurological status*							
Behavior		4.00	0.85	0.15	4.00	0.85	0.15
Irritable crying		4.00	1.00	0.12	4.00	1.00	0.12
Posture: muscle tone, spontaneous movement		5.00	1.00	0.11	5.00	1.00	0.08
* Reflexes, primitive/five reflexes/red reflex, Erb’s palsy and seizure		5.00	0.85	0.15	5.00	0.85	0.13
*Neonatal status*							
* IV site: redness, swelling, edema, clean, and duration of IV insertion		5.00	1.00	0.06	5.00	1.00	0.00
* Fluid management: cc/kg/day, electrolyte management: mg/kg/day		5.00	1.00	0.08	5.00	1.00	0.00
* Blood sugar level		5.00	0.85	0.13	5.00	0.85	0.13
* Intake and output		5.00	1.00	0.08	5.00	1.00	0.06
* Breast feeding frequency and effectiveness		5.00	0.85	0.15	5.00	1.00	0.10
* Vaccination status		5.00	0.69	0.18	5.00	0.85	0.13
Development		5.00	0.69	0.17	5.00	1.00	0.00
Incubator and room temperature		5.00	1.00	0.08	5.00	1.00	0.06
*Maternal status*							
* Body weight, and condition of the mother before and after delivery		4.00	0.38	0.25	4.00	0.38	0.16
* Nutrition, breasts/express breast milk, and colostrum		5.00	0.85	0.15	5.00	0.85	0.15
Drug use, alcohol use, and coping post-partum		5.00	1.00	0.10	5.00	1.00	0.11
**2. Psychological assessment**							
Assess mood of mother/caregiver to identify anxiety/worries/scary/depress		4.00	0.69	0.17	4.00	0.69	0.17
Observe face expression of mother/caregiver to identify feeling		4.00	0.54	0.18	4.00	0.54	0.18
Assess perception and belief of neonatal sickness or issue at home		4.00	0.85	0.13	4.00	0.85	0.12
**3. Social-economic and family assessment**							
Recognizes role of parents in decision making about neonate’s health		4.00	0.54	0.19	4.00	0.54	0.19
Assess whether the family is able to take care of financial issues		4.00	0.38	0.13	4.00	0.38	0.13
Assess neglecting issues of the young mothers from their family		4.00	0.38	0.18	4.00	0.38	0.16
Assess mother’s knowledge in taking care of baby		4.00	0.85	0.15	4.00	0.85	0.14
**B. Nursing diagnosis**							
**1. Actual nursing diagnosis**							
* Hypothermia		5.00	0.85	0.08	5.00	1.00	0.00
Hyperthermia		5.00	1.00	0.08	5.00	1.00	0.00
Ineffective thermoregulation		5.00	0.85	0.14	5.00	0.85	0.11
Airway obstruction		5.00	1.00	0.00	5.00	1.00	0.00
* Impaired gas exchange		5.00	1.00	0.06	5.00	1.00	0.00
Ineffective breathing pattern		5.00	1.00	0.08	5.00	1.00	0.06
* Asphyxia		5.00	1.00	0.06	5.00	1.00	0.00
Pain		5.00	0.85	0.14	5.00	1.00	0.08
Umbilical cord infection		5.00	0.85	0.15	5.00	0.85	0.14
Necrotizing enterocolitis		5.00	1.00	0.06	5.00	1.00	0.00
Neonatal jaundice		5.00	1.00	0.08	5.00	1.00	0.06
Premature/low birth weight infant		5.00	1.00	0.06	5.00	1.00	0.00
Ineffective feeding		5.00	1.00	0.10	5.00	1.00	0.09
Ineffective breast feeding		4.00	0.38	0.22	4.00	0.38	0.20
Interrupt breast feeding		4.00	0.23	0.23	4.00	0.38	0.19
**2. Risk for nursing diagnosis**							
Risk for aspiration		5.00	1.00	0.08	5.00	1.00	0.06
* Risk for infection		5.00	1.00	0.06	5.00	1.00	0.00
Risk for body temperature alteration		5.00	1.00	0.09	5.00	1.00	0.08
Risk for altered nutrition		5.00	0.85	0.13	5.00	0.85	0.13
Risk for fluid volume deficit		5.00	1.00	0.08	5.00	1.00	0.06
Risk for fall down		5.00	1.00	0.11	5.00	1.00	0.11
Risk for sore		4.00	1.00	0.11	4.00	1.00	0.11
**C. Planning**							
* Set safety goals for neonate to overcome actual and risk for nursing diagnosis from admission to discharge		5.00	1.00	0.11	5.00	1.00	0.09
* Provide interventions to fit with actual and risk for nursing diagnosis		5.00	1.00	0.11	5.00	1.00	0.09
**D. Implementation**							
**1. Nursing intervention for ineffective thermo regulation**							
*Reduce or eliminate the sources of heat loss*							
*Evaporation*							
* When a shower, prepare a warm environment		4.00	0.85	0.15	4.00	1.00	0.12
* Wash and dry each section to reduce evaporation		4.00	0.69	0.17	4.00	1.00	0.12
* Limit the time of contact with clothing or a wet blanket		4.00	0.69	0.15	4.00	1.00	0.11
*Convection*							
Avoid the flow of air		4.00	0.54	0.19	4.00	0.54	0.19
*Conduction*							
Warm all goods for care such as stethoscope, scales, hand caregivers, clothes, and bed linen		4.00	0.85	0.15	4.00	1.00	0.11
*Radiation*							
Reduce the objects that absorb heat		4.00	0.38	0.25	4.00	0.69	0.22
*Monitor neonate’s body temperature*							
*If temperature is below normal*							
* Use two blankets		4.00	0.69	0.17	4.00	0.85	0.14
* Wear headgear		4.00	0.85	0.15	4.00	1.00	0.11
* Assess environmental sources for heat loss		5.00	0.85	0.15	5.00	1.00	0.10
* If hypothermia settled >1 h, refer to physician		5.00	0.69	0.22	5.00	0.85	0.19
* Review the complications of cold stress, hypoxia, respiratory acidosis, hypoglycemia, fluid/electrolyte imbalance, and weight loss		5.00	1.00	0.09	5.00	1.00	0.06
*If temperature is above normal*							
* Remove blanket		5.00	1.00	0.11	5.00	1.00	0.09
* Remove headgear, when worn		5.00	1.00	0.11	5.00	1.00	0.10
* Assess environmental temperature again		5.00	1.00	0.10	5.00	1.00	0.09
* If temperature is not reduced to normal >1 h, report to physician		5.00	0.85	0.11	5.00	1.00	0.09
*Teach to measure temperature*							
* Demonstrate how to save heat during bathing		5.00	1.00	0.11	5.00	1.00	0.10
* Teach to measure temperature		5.00	1.00	0.11	5.00	1.00	0.10
* Teach caregiver why neonates are vulnerable to heat and cold weather		5.00	1.00	0.11	5.00	1.00	0.09
* Refer to hypothermia and hyperthermia for prevention		5.00	0.85	0.15	5.00	1.00	0.08
**2. Nursing Interventions for neonate with airway and respiratory problems**							
* Place neonate in semi-follower/comfortable position		5.00	1.00	0.08	5.00	1.00	0.06
* Maintain free airway		5.00	1.00	0.09	5.00	1.00	0.08
Provides oxygen per prescription		5.00	1.00	0.09	5.00	1.00	0.08
* Monitor dyspnea, tachypnea, breath sounds, increased respiratory effort, lung expansion and weakness		5.00	1.00	0.06	5.00	1.00	0.00
* Evaluate changes of level of consciousness, cyanosis, skin color, mucous membranes, and nails		5.00	1.00	0.08	5.00	1.00	0.06
**3. Nursing interventions for neonate with infection**							
Keep neonate in isolation room		5.00	0.85	0.13	5.00	0.85	0.13
Monitor vital signs every 2 h, notify the physician if vital signs are abnormal		5.00	1.00	0.09	5.00	1.00	0.06
Maintain a good temperature for an incubator and room		5.00	0.85	0.14	5.00	0.85	0.13
Wash hands before and after touching the neonate		5.00	1.00	0.00	5.00	1.00	0.00
Make sure that caregivers wash hands before touching/holding neonate		4.00	1.00	0.06	4.00	1.00	0.00
Let neonate rest, avoid holding if unnecessary		5.00	0.85	0.15	5.00	0.85	0.15
Administer antibiotics per prescription (including medication skill)		5.00	1.00	0.08	5.00	1.00	0.00
Surgical patient care		5.00	0.85	0.13	5.00	1.00	0.08
**4. Nursing interventions for impaired skin integrity**							
* Assess skin color every 8 h		5.00	0.85	0.15	5.00	1.00	0.08
* Monitor direct and indirect bilirubin		5.00	1.00	0.11	5.00	1.00	0.09
* Change position every 2 h		5.00	0.85	0.15	5.00	0.85	0.13
* Massage the skin		5.00	0.85	0.15	5.00	0.85	0.13
* Keep clean skin and moisture		5.00	0.85	0.15	5.00	0.85	0.13
Phototherapy		5.00	1.00	0.06	5.00	1.00	0.06
**5. Fluid volume deficit**							
Monitor signs of dehydration such as skin turgor/fontanel/eyes		5.00	1.00	0.08	5.00	1.00	0.06
Monitor intake output		5.00	1.00	0.09	5.00	1.00	0.06
Record the frequency and amount of urine and stools		5.00	1.00	0.08	5.00	1.00	0.06
Monitor fluid and electrolytes balance		4.00	1.00	0.08	4.00	1.00	0.08
Explain the mother to breast feed often		4.00	0.85	0.15	4.00	1.00	0.11
Blood transfusion		4.00	1.00	0.06	5.00	1.00	0.06
Foley insertion		5.00	0.54	0.29	4.00	0.85	0.15
**6. Nursing interventions for interrupted breastfeeding**							
Assess mother’s perception and knowledge about breast feeding		4.00	1.00	0.12	4.00	1.00	0.11
Give emotional support to mother and accept decision regarding cessation/continuation of breast feeding		4.00	0.69	0.17	4.00	0.85	0.13
Demonstrate use of manual breast pump		4.00	0.08	0.28	4.00	0.23	0.30
Explain techniques for storage of expressed breast milk		4.00	0.54	0.24	4.00	0.69	0.21
Provide privacy, calm surroundings when mother breast feeds		4.00	0.38	0.24	4.00	0.69	0.20
Recommend for infant sucking on a regular basis		4.00	0.54	0.23	4.00	0.54	0.21
Encourage mother to obtain adequate rest, maintain fluid and nutritional intake, and schedule breast pumping every 3 h while awake		5.00	0.38	0.24	5.00	0.69	0.21
**7. Nursing interventions: risk for altered nutrition**							
Weight neonate in gram daily		5.00	1.00	0.09	5.00	1.00	0.00
Assess maturity reflex, with regard to feeding such as sucking, swallowing and cough		5.00	1.00	0.06	5.00	1.00	0.00
Monitor input and output and calculate consumption of calories and electrolytes daily		5.00	1.00	0.08	5.00	1.00	0.06
Assess level of hydration, note fontanel, skin turgor, urine-specific gravity, condition of mucous membranes, and weight fluctuations		5.00	1.00	0.09	5.00	1.00	0.06
Assess signs of poor feeding, nervous, crying high tone, trembling, eyes upside down and seizure activity		5.00	1.00	0.06	5.00	0.85	0.11
* Administer TPN(Total Parenteral Nutrition) per prescription (including medication skill)		4.00	0.85	0.11	4.00	1.00	0.06
Enema		5.00	0.85	0.15	5.00	0.85	0.13
**8. Nursing interventions for pain**							
Encourage mother to provide breast feeding		5.00	0.69	0.21	5.00	0.85	0.14
Repositioning, swaddling, and nesting		5.00	1.00	0.11	5.00	0.85	0.13
Facilitated tucking and containment holding		4.00	0.85	0.15	4.00	0.85	0.13
Decreasing environmental sensors		4.00	0.54	0.19	4.00	0.69	0.16
Change nappy as needed		5.00	0.69	0.17	5.00	1.00	0.10
Allowing neonate to grasp a finger		5.00	0.85	0.15	5.00	1.00	0.11
Kangaroo care		5.00	0.85	0.14	5.00	1.00	0.09
**E. Evaluation**							
The neonate requiring intervention is promptly identified and is started early		5.00	0.85	0.15	5	0.85	0.14
The neonate’s metabolic and physiologic processes are stabilized, and recovery is proceeding without complications		5.00	0.54	0.20	5	1.00	0.06
Infant maintains temperature at 36.5 °C to 37 °C		5.00	0.69	0.17	5.00	1.00	0.08
Neonate maintains a respiratory rate of 30–60 breaths per minute		5.00	1.00	0.08	5.00	1.00	0.08
Neonate will exhibit no signs of infection		5.00	0.85	0.14	5.00	0.85	0.13
Fluid volume will be maintained: Oral mucosa moist and pink, skin turgor elastic, urine output at least 1–2 mL/kg/hr.		5.00	1.00	0.10	5.00	1.00	0.09
Neonate will maintain adequate nutritional intake: Weight gain or maintenance occurs. Consumes adequate diet for age		5.00	1.00	0.11	5.00	0.85	0.13
Neonate will be in comfort and free from pain		4.00	0.69	0.18	5.00	1.00	0.10
**F. Ethics**							
Advocate for equitable to healthcare consumer		5.00	0.69	0.17	4.00	0.85	0.15
Provide care follow guidelines/protocols so that the care nurse provides are safe for neonate		5.00	1.00	0.08	5.00	1.00	0.06
Maintain confidentiality of data, plans, and results to protect the dignity of high-risk newborns and the privacy of their parents.		5.00	1.00	0.06	5.00	1.00	0.00
Provide care without bias and make ethical decisions while considering the individuality of high-risk neonates.		5.00	1.00	0.06	5.00	1.00	0.00
Recognize parents’ rights to enable parents of high-risk newborns to make choices related to treatment and care.		5.00	1.00	0.09	5.00	1.00	0.08
**G. Performance Evaluation**							
Evaluate the outcomes of high-risk neonatal care in accordance with professional practice standards and applicable legislation.		5.00	0.69	0.17	5.00	0.69	0.17
Through evaluation, the contents of high-risk neonatal care are continuously revised and supplemented.		5.00	0.69	0.17	5.00	0.69	0.17
**H1. Education (Health teaching and health promotion)**							
Explain to family about treatment and procedures and follow-up		5.00	1.00	0.11	5.00	1.00	0.11
Tech parents about basic health information	Nutrition/breast feeding	4.00	1.00	0.11	5.00	1.00	0.11
	Reproductive health	4.00	0.69	0.17	4.00	0.85	0.14
	Body hygiene	5.00	0.85	0.15	4.00	1.00	0.12
	Hand hygiene correctly	5.00	1.00	0.09	5.00	1.00	0.09
	Prevent hypothermia	5.00	0.85	0.15	5.00	1.00	0.09
	Recognize signs of sick neonate	5.00	1.00	0.08	5.00	1.00	0.06
	Schedule of vaccination and immunization	5.00	1.00	0.09	5.00	1.00	0.06
	Prevention of infection	5.00	1.00	0.09	5.00	1.00	0.06
	Umbilical cord care	5.00	1.00	0.09	5.00	1.00	0.08
	Human contact	5.00	0.85	0.26	5.00	0.85	0.15
	Discharge	5.00	1.00	0.10	5.00	1.00	0.09
**H2. Education (Continuing Education)**							
Participate in nursing education as appropriate to educational level and position		5.00	0.85	0.15	5.00	0.85	0.13
Participate in neonatal nursing training to update knowledge and competencies		5.00	1.00	0.10	5.00	1.00	0.08
Conduct self-directed learning, read textbooks, and search internet		5.00	0.85	0.20	5.00	0.85	0.19
**I. Evidence-based practice and research**							
Develop knowledge from routine jobs toward research work that would apply to nursing practice		5.00	1.00	0.11	5.00	1.00	0.10
Introduce important research finding and evidence-based practice to other nurses		5.00	1.00	0.11	5.00	1.00	0.10
Utilizes evidence-based practice and research finding to guide practice		5.00	1.00	0.11	5.00	1.00	0.09
Participate in nursing research according to educational level/role		5.00	0.85	0.15	5.00	1.00	0.10
Integrates research findings into the development of guidelines and standards of care		5.00	0.85	0.14	5.00	0.85	0.13
**J. Consultation and cooperation**							
Make effective communication with families and members of healthcare team		5.00	0.69	0.18	5.00	1.00	0.10
Participate in the health management team’s consultation of high-risk neonatal care		5.00	0.69	0.31	5.00	1.00	0.08
Consult a specialist or an external institution if needed		5.00	0.69	0.22	5.00	1.00	0.09
Form a support network with nurses and other colleagues to ensure the smooth delivery of care		5.00	1.00	0.08	5.00	1.00	0.06
Cooperate with the high-risk neonatal health care team to jointly perform tasks		5.00	0.69	0.22	5.00	0.85	0.13
**K. Use of resources**							
Identify human and material resources and skills to manage critical situations associated with high-risk newborns		5.00	0.69	0.18	5.00	0.85	0.15
Identify and manage human and material resources in a cost-effective manner		5.00	0.69	0.18	5.00	0.69	0.18
Identify and link local community resources to manage critical situations associated with high-risk newborns		5.00	0.54	0.24	5.00	0.69	0.18
Manage various documents related to high-risk newborns		5.00	0.38	0.22	5.00	0.54	0.20
Use the information related to high-risk newborns effectively		5.00	0.69	0.17	5.00	0.69	0.17
**L. Quality management in nursing**							
Monitor and improve the quality of high-risk neonatal care		5.00	0.85	0.14	5.00	0.85	0.14
Participate in health care policymaking related to high-risk neonatal health problems		4.00	0.38	0.27	4.00	0.69	0.28
Participate in identifying, finding, and implementing solutions related to high-risk neonatal health problems in the community and beyond		5.00	0.38	0.29	5.00	0.38	0.29
Evaluate high-risk neonatal care records		5.00	0.69	0.17	5.00	0.69	0.17

CVR = content validity ratio * alternative practice in the school grounds.
